# Supplementary material for: The Angiotensin II Receptor Blocker Losartan Sensitizes Human Liver Cancer Cells to Lenvatinib-Mediated Cytostatic and Angiostatic Effects
Source: Cells. 2021 Mar 5;10(3):575. doi: 10.3390/cells10030575 (PMC8001516; doi:10.3390/cells10030575)
Supplement: Supplementary file 1 [file cells-10-00575-s001.pdf]

Supplementary Materials

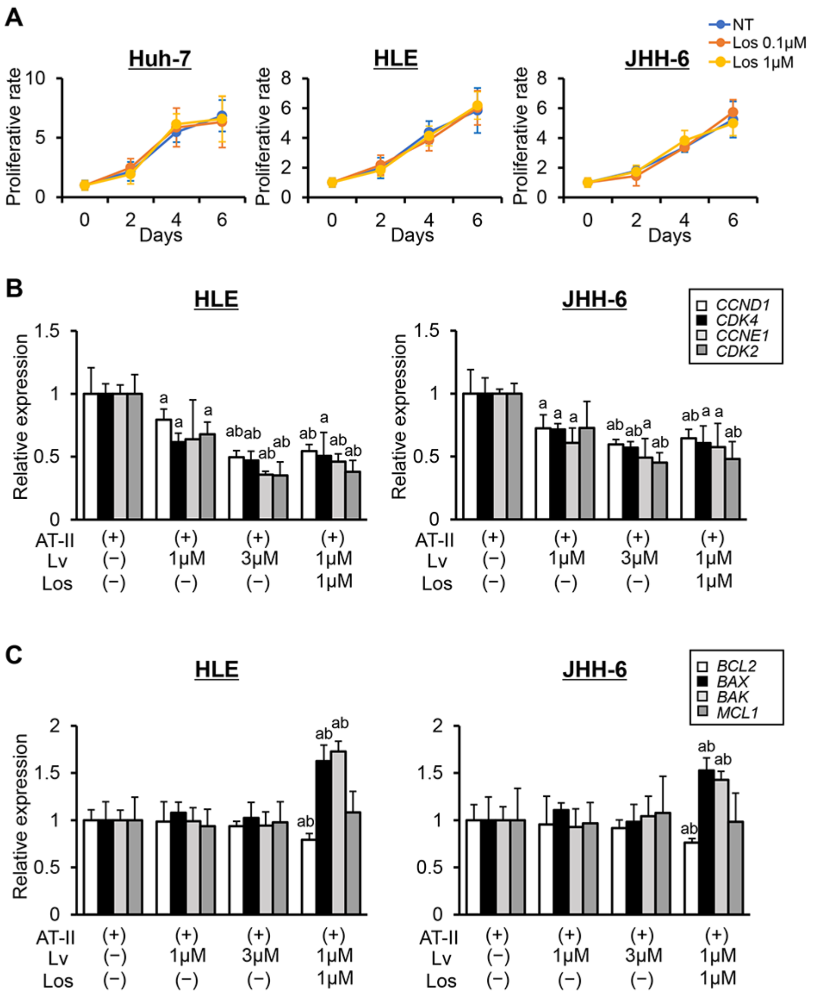

Figure S1. *In vitro* cytostatic effects of lenvatinib and losartan on different HCC cell lines**.** (**A**) Cell proliferation of human liver cancer cells (Huh-7, HLE and JHH-6) incubated with losartan (Los) (0, 0.1 and 1 μM) under the condition without stimulation of angiotensin-II (AT-II) for 0-6 days. (**B** and **C**) Relative mRNA expression levels of cell cycle-related markers (**B**) and apoptosis-related markers (**C**) in HLE and JHH-6. Cells were pre-treated with AT-II (1 μM) for 12 h and subsequently treated with Los (1 μM) and/or lenvatinib (Lv) (1 or 3 μM) for 12 h. The mRNA expression levels were measured by qRT-PCR, and *GAPDH* was used as internal control. Quantitative values are relatively indicated as fold changes to the values of (**A**) group at the start of treatment with losartan in each dose, (**B** and **C**) group of AT-II(+)/Lv(-)/Los(-). Data are mean ± SD (n = 3 independent experiments with n = 8 samples per condition). [a] $p < 0.05$, [b] $p < 0.05$ compared with group treated with AT-II(+)/Lv(-)/Los(-) and AT-II(+)/Lv(1 μM)/Los(-), respectively (**B** and **C**).

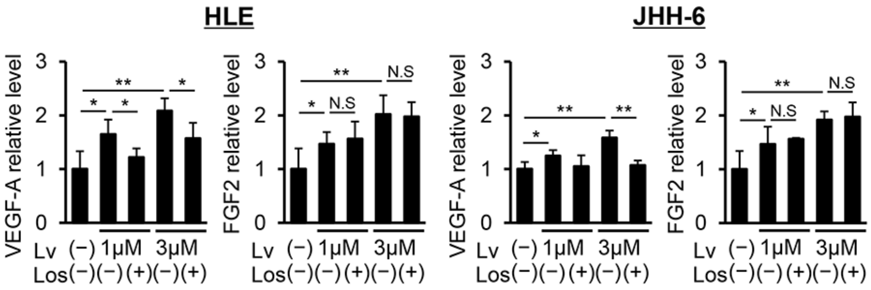

Figure S2. *In vitro* VEGF-A and FGF2 levels in different HCC cell lines treated with lenvatinib and losartan. VEGF-A and FGF2 levels in HLE or JHH-6-cultured media. Cells were treated with Los (1 μM) and/or lenvatinib (Lv) (1 or 3 μM) for 24 h. Quantitative values are relatively indicated as fold changes to the values of group of Los(-)/Lv(-). Quantitative values are relatively indicated as fold changes to the values of group of Los(-)/Lv(-). Data are mean ± SD (n = 3 independent experiments with n = 8 samples per condition). * $p < 0.05$; ** $p < 0.01$ indicating a significant difference between groups N.S: Not significant.

Table S1. List of primers used in q-PCR.

gene	Sense (5′-3′)	Antisense (5′-3′)
	**Mouse**	
CD34	GGGTAGCTCTCTGCCTGATG	TCTCTGAGATGGCTGGTGTG
	**Human**	
CCND1	CCCTCGGTGTCCTACTTCAA	CTTAGAGGCCACGAACATGC
CDK4	CCCACACAAGCGAATCTCTG	ACCCTCCATAGCCTCAGAGA
CCNE1	CGCTGATGAAGATGCACACA	ACAGAAGAGAACGTGGAGCA
CDK2	AGGCATGAGGAATCTGGGAG	GAGGTGGACGTCAGAGGAAA
BCL2	CCACGTGGTAAGATCCTCCA	AGAGGCTGGGCACATTTACT
BAX	AACATGGAGCTGCAGAGGAT	CCAATGTCCAGCCCATGATG
BAK	CCAGGACACAGAGGAGGTTT	CTCTGAGTCATAGCGTCGGT
MCL1	AGTAGGAGCTGGTTTGGCAT	TGCTTTTCTGGCTAGGTTGC
VEGF-A	GGGCAGAATCATCACGAAGT	TGGTGATGTTGGACTCCTCA
FGF2	CATGGCTGCAGTTCCTTTGT	TCCTGCCACACAAATTTGCA
CXCL8	CAGTTTTGCCAAGGAGTGCT	ACTTCTCCACAACCCTCTGC
